# Connexin 43 Suppresses Tumor Angiogenesis by Down-Regulation of Vascular Endothelial Growth Factor via Hypoxic-Induced Factor-1α

**DOI:** 10.3390/ijms16010439

**Published:** 2014-12-26

**Authors:** Wei-Kuang Wang, Man-Chin Chen, Hon-Fai Leong, Yu-Liang Kuo, Chun-Yu Kuo, Che-Hsin Lee

**Affiliations:** 1 Department of Environmental Engineering and Science, Feng Chia University, Taichung 40407, Taiwan; E-Mail: wkwang@fcu.edu.tw; 2 Graduate Institute of Basic Medical Science, School of Medicine, China Medical University, Taichung 40404, Taiwan; E-Mails: menny628@hotmail.com (M.-C.C.); u100004422@cmu.edu.tw (C.-Y.K.); 3 Department of Microbiology, School of Medicine, China Medical University, Taichung 40404, Taiwan; E-Mail: isaac_leong725@hotmail.com; 4 School of Medical Imaging and Radiological Sciences, Chung Shan Medical University, Taichung 40402, Taiwan; E-Mail: yuliangkuo@mercury.csmu.edu.tw; 5 Department of Medical Imaging, Chung Shan Medical University Hospital, Taichung 40402, Taiwan

**Keywords:** connexin 43 (Cx43), vascular endothelial growth factor (VEGF), hypoxic-induced factor-1α (HIF-1α), angiogenesis

## Abstract

Previous work showed that connexin 43 (Cx43) reduced the expression of hypoxic-induced factor-1α (HIF-1α) in astrocytes. HIF-1α is a master transcription factor for angiogenesis in tumor. Angiogenesis is essential for tumor progression. Here, we investigated the role of Cx43 in vascular endothelial growth factor (VEGF) production and angiogenesis in murine tumor. In the study, mouse B16F10 and 4T1 cells were overexpressed or knockdown with Cx43. The expression profiles as well as activity of the treated cells were examined. Furthermore, reduced Cx43 expression in B16F10 and 4T1 cells causes increased expression of VEGF and enhanced the proliferation of endothelial cells. On the contrary, the expression of VEGF and the proliferation of endothelial were increased in the conditioned medium of *Cx43*-knockdown tumor cells. We subcutaneously transplanted *Cx43*-overexpressing B16F10 cells into mice to evaluate the roles of Cx43 in the tumor angiogenesis. Both tumor size and the number of vessels growing in the tumor were markedly decreased compare with control group. Our findings suggest that Cx43 inhibited tumor growth by reducing angiogenesis.

## 1. Introduction

Gap junctions mediate cell communication by allowing the passage of molecules from one cell to another. The major role of gap junction intercellular communication (GJIC) is considered to be the maintenance of homeostasis in organisms [1]. Gap junctions are formed by two hemichannels, called connexons, each made of six connexin (Cx) proteins. Cx43 is ubiquitous and reduced in a variety of tumor cells [2,3,4,5]. Cx43 may influence the response of tumor cells to treatments by facilitating the passage of antitumor drugs or death signals between neighboring tumor cells [6]. Many tumor cells are characterized by dysfunction of Cx43. Cx43 has been identified as tumor suppressor or enhancer, a difference that appears to be dependent on the type and stage of tumor [7]. A hypoxic microenvironment is characteristic of many solid tumors. Hypoxia is also associated with a more malignant phenotype, affecting genomic stability, apoptosis, angiogenesis and metastasis [8]. Induction of angiogenesis plays an important role in the development and progression of most human tumors, including mammary tumor and melanoma [9,10]. Hypoxia-inducible factor (HIF) is a heterodimeric transcription factor that mediates responses to hypoxia by binding to hypoxia-response elements (HRE) present within target genes. The HIF-1 transcription factors are composed of oxygen-sensitive HIF-1α subunit and a constitutively expressed HIF-1β subunit. Previous studies showed that overexpression of HIF-1α in various tumor types compared to the respective normal tissues, including brain, colon, breast, gastric, lung, skin, ovarian, prostate, and renal carcinomas [8,11]. Therapeutic targeting of angiogenesis has recently been explored to inhibit malignant tumor growth and metastasis [12,13]. Previously, Cx43 has been able to reduce angiogenesis in breast cancer [9]. However, the role of Cx43 in tumor angiogenesis and the mechanism of Cx43-induced antiangiogenesis in tumors are less defined. In the present work, we sought to identify the signaling pathway responsible for this process in breast tumor and melanoma.

## 2. Results

### 2.1. Murine Tumor Cells Express Cx43 and HIF-1α

**Figure 1:**
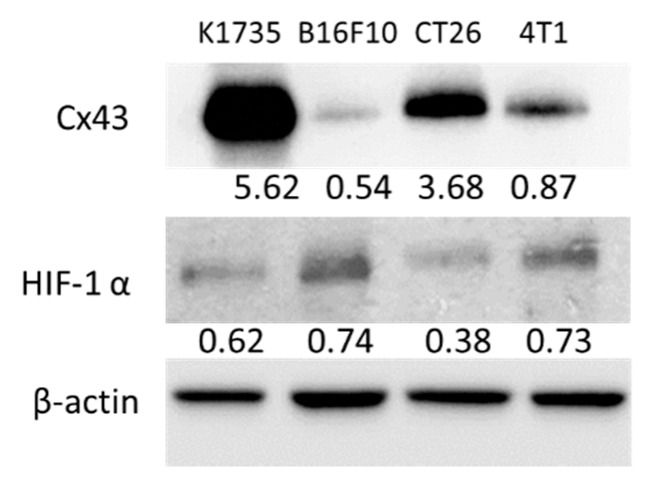
Murine tumor cells express connexin 43 (Cx43) and hypoxic-induced factor-1α (HIF-1α). The expression of Cx43 and HIF-1α was measured by Western blot analysis. β-actin expression served as loading controls and total protein. Inserted values indicated relative protein expression in comparison with β-actin.

Cx43 have been identified as tumor suppressors, and interact with various intracellular protein compared with other Cx [7]. This study aims to identify the signal pathway of Cx43 involved in tumor angiogenesis. First, we identified the expression pattern of Cx43 in a variety of murine tumor cells. Furthermore, to investigate whether Cx43 and HIF-1α are expressed in cultured murine tumor cell lines, we first performed immunoblotting assay. The result indicated that murine tumor cell lines (Murine K1735 melanoma [6], murine melanoma B16F10 [12], murine breast cancer 4T1 [14], and murine CT26 colon cancer [15]) express Cx43 and HIF-1α (Figure 1). The fact that expression of Cx43 considerably varies in different cells and that HIF-1α can be induced simultaneously with Cx43 [16] raises the question of whether there exists a link between the HIF-1α transcriptional activity and Cx43. The expression of Cx43 in B16F10 and 4T1 cells was much lower than those in K1735 and CT26 cells, respectively, as determined by immunoblotting assay. Conversely, B16F10 and 4T1 cells displayed higher levels of HIF-1α protein expressions than K1735 and CT26 cells, respectively. Taken together, these results may suggest an inverse correlation between the protein expression of Cx43 and HIF-1α in murine tumor cells.

### 2.2. Cx43 Regulates the Expression of HIF-1α

**Figure 2:**
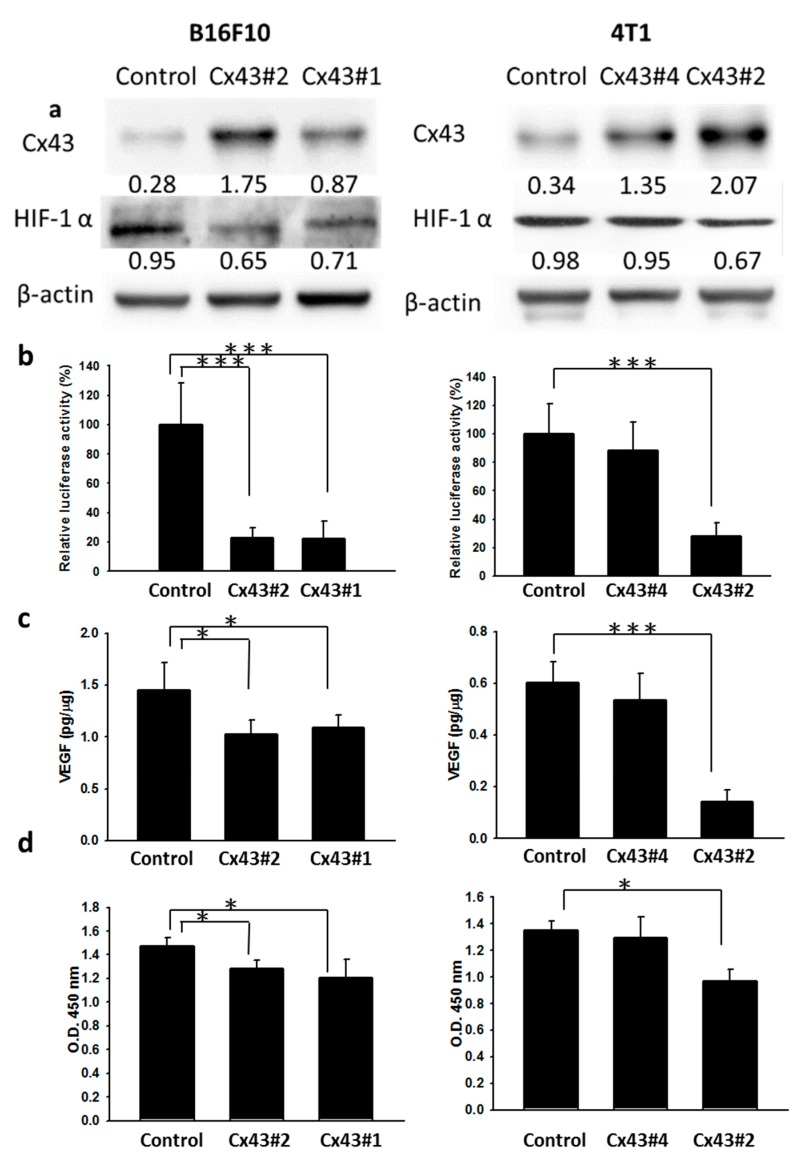
*Cx43*-overexpressing cells regulate the expression of HIF-1α, vascular endothelial growth factor (VEGF), and the proliferation of endothelial cells. (**a**) The expression of Cx43 and HIF-1α was measured in *Cx43*-overexpressing cells by Western blot analysis. β-actin expression served as loading controls for and total protein. Inserted values indicated relative proteins expression in comparison with β-actin; (**b**) *Cx43-*overexpressing cells were cotransfected with pCLNCX-6× HRELuc and pTCYLacZ plasmids. At 6 h post-transfection, their luciferase activities were determined and normalized with β-gal activity. Data shown were the mean ± SD (*n* = 4); (**c**) The conditioned medium of *Cx43*-overexpressing cells was measured by ELISA. Data shown were the mean ± SD (*n* = 4); and (**d**) The conditioned medium of *Cx43*-overexpressing cells reduced the proliferation of endothelial cells. The HEMC-1 cells treated with conditioned medium of *Cx43*-overexpressing cells were examined the proliferation activity. Cell viability was assessed by the WST-1 assay. Data shown were the mean ± SD (*n* = 4). * *p* < 0.05; *** *p* < 0.001.

To determine whether the HIF-1α expression was regulated by Cx43, the *Cx43*-overexpressing cell clones were selected including B16F10 and 4T1 cells. Figure 2a showed that the expression of HIF-1α was decreased in *Cx43*-overexpressing tumor cells (B16F10 and 4T1). The extent of hypoxic responsiveness of hypoxia-response element (HRE) reporter assay in different tumor cells varied, ranging from 0.2–0.3-fold compared with control cells (Figure 2b). The levels of hypoxic responsiveness were higher in B16F10 and 4T1 control cells than in *Cx43*-overexpressing cells. Since hypoxia and HIF-1α transactivate vascular endothelial growth factor (VEGF), a growth factor which can induce angiogenesis in tumor growth [17]. VEGF expression in the tumor cells was measured. As shown in Figure 2c, the protein levels of VEGF were dramatically decreased in *Cx43*-overexpressing cells. Collectively, Cx43 decreases transcriptional activity of HIF-1α and inhibits the expression of VEGF in tumor cells. The human microvascular endothelial cells (HMEC-1) treated with those conditioned medium of cells were tested for their ability to inhibit the proliferation of endothelial cells. Figure 2d demonstrates that proliferation of HMEC-1 cells was decreased upon addition of conditioned medium of *Cx43*-overexpressing cells compared with that from control cells. The Cx43#4 (*Cx43*-overexpressing stable clone number 4) 4T1 cells that express only modest amounts of Cx43 did not significantly inhibit HIF-1α expression, its transcriptional activities, VEGF expression and endothelial proliferation.

### 2.3. Reduction of Cx43 Expression by Cx43: Short Hairpin RNA (shRNA) Tumor Cells Are More Hypoxia-Responsive by Increasing the Transcriptional Activity and Stability of HIF-1α Protein

**Figure 3:**
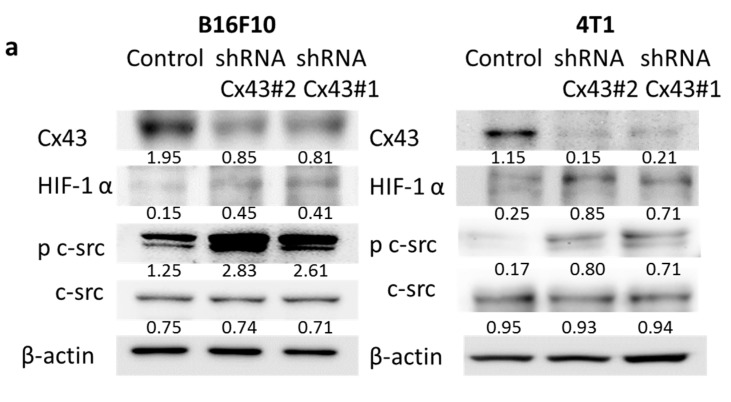

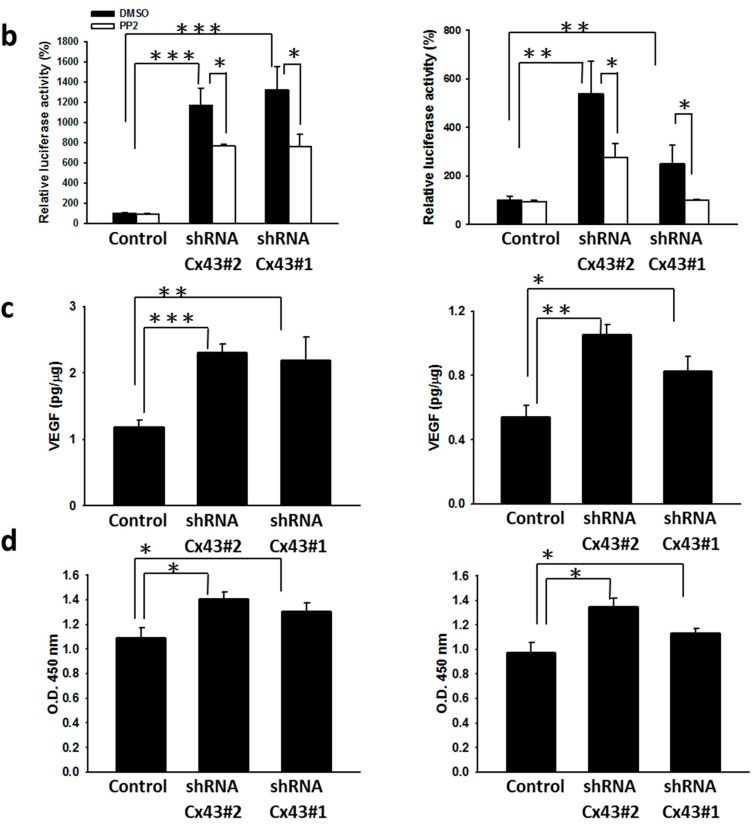
*Cx43*-knockdown cells regulate the expression of HIF-1α, c-Src, VEGF and the proliferation of endothelial cells. **(a**) The expression of Cx43, c-Src, and HIF-1α was measured in *Cx43*-knocknown cells by Western blot analysis. β-actin expression served as loading controls for and total protein. Inserted values indicated relative protein expression in comparison with β-actin; (**b**) *Cx43-*knockdown cells were cotransfected with pCLNCX-6× HRELuc and pTCYLacZ plasmids. Then, cells were treated with 100 ng/μL PP2 or DMSO for 1 h. At 6 h post-transfection, their luciferase activities were determined and normalized with β-gal activity. Data shown were the mean ± SD (*n* = 4); (**c**) The conditioned medium of *Cx43*-knockdown cells was measured by ELISA. Data shown were the mean ± SD (*n* = 4); (**d**) The conditioned medium of *Cx43-*knockdown cells reduced the proliferation of endothelial cells. The HEMC-1 cells treated with conditioned medium of *Cx43-*knockdown cells were examined for proliferation activity. Cell viability was assessed by the WST-1 assay. Data shown were the mean ± SD (*n* = 4). * *p* < 0.05; ** *p* < 0.01; *** *p* < 0.001.

Because Cx43 can influence HIF-1α protein expression and thereby abrogate HIF-1α-mediated transcriptional activity [16], we examined whether the difference in the HIF-1α protein expression and HIF-1α transcriptional activity between Cx43 shRNA-transfected and control vector-transfected tumor cells could influence HIF-1α transcriptional activity. First, we examined the protein expression of Cx43 and HIF-1α in *Cx43*-knockdown cells by immunoblotting assay (Figure 3a). As expected, Cx43 was down-regulated in the Cx43 shRNA-transfected cells. By contrast, the expression of HIF-1α was dramatically increased in Cx43 shRNA-transfected cells. To gain insights into the role of Cx43 on HIF-1α transcriptional activity, we used Cx43 shRNA stable transfectants derived from B16F10 and 4T1 cells to study the correlation between Cx43 and HIF-1α transcriptional activities. Figure 3b shows that B16F10 cells bearing Cx43 expression, when transfected with Cx43 shRNA, decreased Cx43 expression (Figure 3a), and concomitantly increased HIF-1α transcriptional activity (Figure 3b). Similarly, 4T1 cell transfectants exhibited lower Cx43 expression compared with their control counterpart (Figure 3a). Accordingly, reducing Cx43 expression by Cx43 shRNA rendered 4T1 cells more responsive to HIF-1α transcriptional activity (Figure 3b). The intracellular *C*-terminal domain of Cx43 inhibits the non-receptor tyrosine kinase c-Src activity and c-Src can activate HIF-1α [16]. In this study, we showed that c-Src is activated by silencing Cx43 (Figure 3a). In order to investigate whether c-Src was also involved in this signal pathway, the activity of c-Src was inhibited by PP2, and HIF-1α transcriptional activity was measured (Figure 3b). The HIF-1α transcriptional activity was significantly reduced after PP2 treatment in Cx43 shRNA-transfected cells compared with control groups (Figure 3b). Consequently, the secretion of VEGF in Cx43 shRNA-transfected cells was significantly increased, and stimulated the proliferation of endothelial cells (Figure 3c,d). Our results point out that down-regulation of Cx43 activates c-Src, which induces HIF-1α expression leading to the secretion of VEGF. Taken together, these results indicate that reducing Cx43 expression contributed to the amplification of HIF-1α-dependent responses. Collectively, these studies suggest that Cx43 expression inhibits the secretion of VEGF, resulting in inhibition angiogenic processes.

### 2.4. Inhibition of Tumor Angiogenesis in Cx43-Overexpressing Tumor Cells

**Figure 4:**
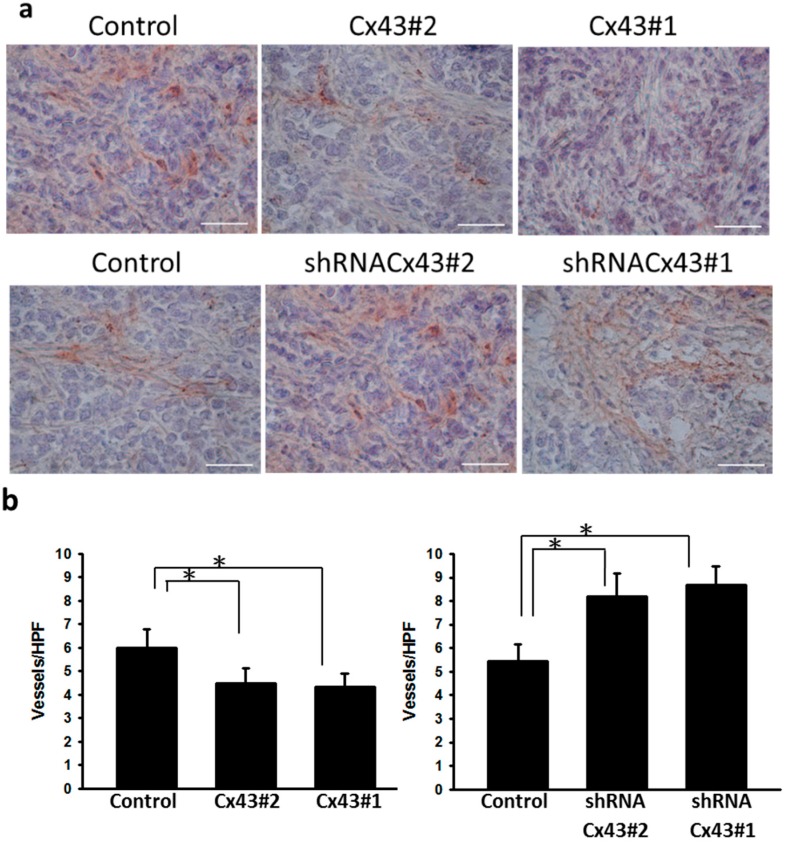
Cx43 reduced the vessel density. Groups of mice were inoculated with *Cx43*-overexpressing B16F10 or *Cx43*-knockdown B16F10 cells (1 × 10^6^) at day 0. Mice were sacrificed at day 16. (**a**) Tumors were excised at day 16, snap frozen and immunostained with rabbit antibody against factor VIII-related antigen (×400); and (**b**) Intratumoral microvessel density was determined by averaging the number of vessels in three areas of highest vessel density at ×400 magnification in each section. Scale bar = 100 μm. Data shown were the mean ± SD (*n* = 4); * *p* < 0.05.

**Figure 5:**
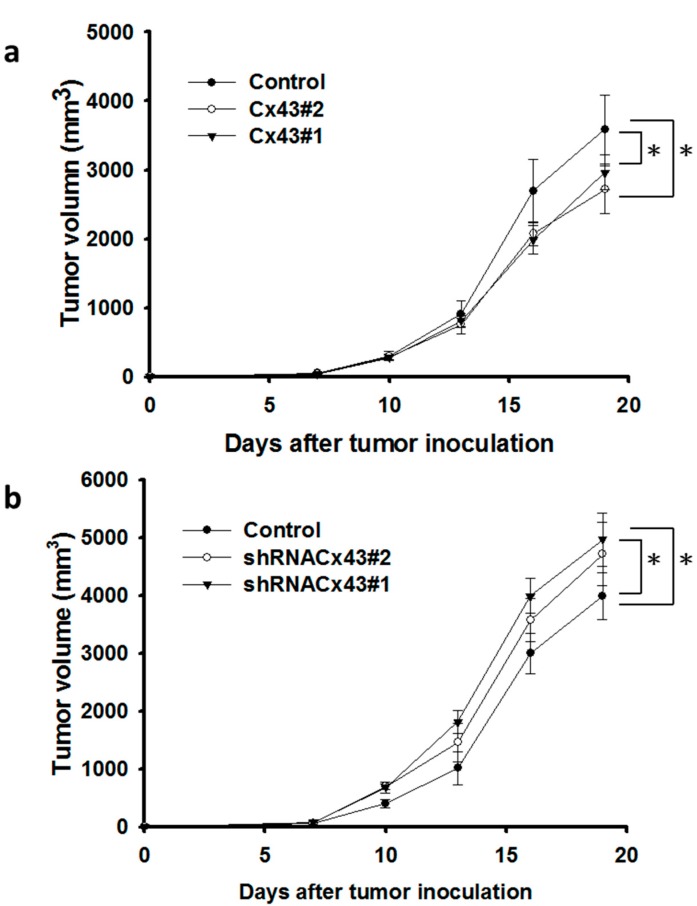
Cx43 retarded tumor growth. Groups of mice were inoculated with *Cx43*-overexpressing B16F10 (**a**) or *Cx43*-knockdown B16F10 cells (1 × 10^6^) (**b**) at day 0. Tumor volumes among different groups were compared at day 19. Data shown were the mean ± SEM (*n* = 4); * *p* < 0.05.

Microvessel density within tumors from B16F10 tumor-bearing mice were analyzed at 16 days after tumor inoculation by immunohistochemistry. The results of immunohistochemical staining are given in Figure 4a. Tumors from *Cx43-*overexpressing mice appeared much less vascularized than their control counterparts, whereas no such difference was found between *Cx43-*overexpressing control and *Cx43-*knockdown control groups (Figure 4b). Furthermore, microvessel density was apparently increased in the tumors of *Cx43-*knockdown group compared with the control groups (Figure 4b). Antitumor effects of Cx43 were evaluated in terms of tumor growth in mice bearing *Cx43-*overexpressing B16F10 melanoma or *Cx43-*knockdown B16F10 melanoma. Figure 5 shows that tumor growth was significantly retarded in mice with *Cx43-*overexpressing cells compared with control mice (Figure 5a). By contrast, the tumor growth was slightly increased in *Cx43-*knockdown B16F10 cell in mice (Figure 5b). Taken together, Cx43 expression vector exerted antitumor effects in melanoma tumor model.

## 3. Discussion

HIF-1α is frequently overexpressed in common human cancers and there is a statistically significant correlation between the presence of mutant tumor suppressor gene and HIF-1α overexpression [18]. Thus, increased HIF-1α activity resulting from loss of tumor suppressor gene function may also contribute to the overexpression of VEGF, which is observed in a wide variety of human cancers [19]. Cells grown in hypoxic environments have higher levels of HIF-1-dependent transcription and lower levels of p53-dependent transcription [20]. It has been shown that p53 inhibits hypoxia-induced HIF-1α expression by facilitating its ubiquitination and subsequent degradation [20]. Similarly, we demonstrate that tumor cells with lower Cx43 expression exhibited higher HIF-1α-dependent gene expression. To further elucidate the relationship between Cx43 and HIF-1α, we used Cx43 shRNA to inhibit the Cx43 expression. Using Cx43 shRNA stable transfectants whose Cx43 expression was reduced, we demonstrate that abrogation of Cx43 expression resulted in increased HIF-1α expression and transcriptional activity in tumor cells. Regarding the function of Cx43 in inhibition tumor growth, a truncated Cx43 could not form gap junctions but inhibited the tumor growth [21], describing that Cx43 inhibited tumor growth via a GJIC-independent mechanism [22]. Meanwhile, Cx43 regulates Src kinase through interaction of the Cx43 *C*-terminal region, irrespective of GJIC function [23]. Expression of Cx43 has been shown to reduce various tumor growths [4,5]. Cx43 inhibits glioblastomas through GJIC-independent mechanism [4]. Previously, we also demonstrated that Cx43 enhanced the antitumor effect of chemotherapy by GJIC function [24]. The tumor suppressive properties of Cx43 are specific to different tumor types, involving both GJIC-dependent and independent mechanisms.

Because Cx43 can inhibit HIF-1α-mediated transcriptional activation, the difference in hypoxic responsiveness in tumor cells may be correlated with their Cx43 status. Our observations indicate that down-regulation of Cx43 expression via Cx43 shRNA contributed to the transcriptional activity of HIF-1α and enhanced VEGF secretion. Histological results also showed that the reduced microvessel density in *Cx43-*overexpression tumor. Our results suggest that Cx43 may be a useful target for treating solid tumors by down-regulation of tumor angiogenesis [25].

## 4. Experimental Section

### 4.1. Cell Lines, Reagents, Mice and Plasmid

Murine B16F10 melanoma, murine K1735 melanoma, murine CT26 colon and murine 4T1 mammary carcinoma were cultured in Dulbecco’s modified Eagle’s medium (DMEM) supplemented with 50 μg/mL gentamicin, 2 mM l-glutamine, and 10% heat-inactivated fetal bovine serum (FBS) at 37 °C in 5% CO_2_. Murine K1735 cells were kindly provided by Dr. M.C. Hung (The University of Texas MD Anderson Cancer Center, San Antonio, TX, USA). Murine 4T1 cells were kindly provided by Dr. W.W. Chang (The Chung Shan Medical University, Taichung, Taiwan). Human HMEC-1 microvascular endothelial cells [12,13] were cultured in EGM endothelial growth medium (Cambrex, East Rutherford, NJ, USA). The c-Src inhibitor, PP2, and DMSO were purchased from Sigma-Aldrich (Sigma-Aldrich, St. Louis, MO, USA). Male C57BL/6 mice at the age of 6–8 weeks were obtained from the Taiwan National Laboratory Animal Center. The animals were maintained in specific pathogen-free animal care facility under isothermal conditions with regular photoperiods. The experimental protocol adhered to the rules of the Animal Protection Act of Taiwan, and was approved by the Laboratory Animal Care and Use Committee. The 24-bp HRE (5'-CAC ACG TGG GTT CCC GCA CGT CCG-3') of the *human lactic dehydrogenase A* gene was obtained by polymerase chain reaction, and six copies of this fragment were individually and in tandem ligated to the 5'-region of the CMV minimal (CMVmini) promoter derived from pTRE vector (Clontech, Palo Alto, CA, USA) at the StuI/EcoRI sites. To construct luciferase reporter plasmids, six copies of HRE ligated to the CMV minimal promoter (6× HRE/CMVmini) were excised from the pTRE-based plasmids by digestion with *Kpn*I and *Hind*III and subcloned into pGL3 (Promega, Madison, WI, USA) at the *Kpn*I/*Hind*III sites [8].

### 4.2. Knockdown of Cx43 and Cx43 Overexpression

The specific siRNA oligos of Cx43 or negative control siRNA oligos was purchased from Invitrogene (Carlsbad, CA, USA). The siRNA oligos of Cx43 designed to knockdown gene expression and the target sequences were listed below: Cx43-siRNA73: CCTGCTGATCCAGTGGTACATCTAT and Cx43-siRNA94: GCGTGAAGGGAAGAAGCGATCCTTA. Lipofectamine RNAiMax reagent (Invitrogene) was used for siRNA transfection following the manufacturer’s protocol. Cx43 shRNA or control vector was purchased from Santa Cruz Biotechnology (Santa Cruz Biotechnology Inc., Santa Cruz, CA, USA).To knockdown *Cx43*, cells were transfected with Cx43 shRNA by lipofectamine 2000 (Invitrogene). Clonal derivatives were isolated by G418 (400 μg/mL) selection and expanded to independent clones, creating shRNACx43#1 B16F10, shRNACx43#2 B16F10 cells, shRNACx43#2 4T1, and shRNACx43#1 4T1. To overexpress Cx43, cells were transfected with pcDNA-Cx43 by lipofectamine 2000. Clonal derivatives were isolated by G418 (400 μg/mL) selection and expanded to independent clones, creating Cx43#1 B16F10, Cx43#2 B16F10 cells, Cx43#2 4T1, and Cx43#4 4T1. Cx43 plasmid was kindly provided by Dr. Shuan-Yow Li (Chung Shan Medical University, Taichung, Taiwan).

### 4.3. Analysis of Hypoxia-Inducible Transcriptional Activities

Various cells grown in 24-well plates were cotransfected with luciferase reporter plasmids driven by HRE promoters (0.66 μg) and pTCYLacZ (0.34 μg), a β-galactosidase (β-gal) expression plasmid driven by the β-actin promoter, by lipofectamine 2000. At 6 h post-transfection, cell lysates were harvested 16 h later. The cell lysates were assessed for their luciferase activities determined by a dual-light luciferase and β-gal reporter gene assay system (Applied Biosystem, Foster City, CA, USA) using a luminometer (Minilumate LB9506, Bad Wildbad, Germany). Relative luciferase activity was measured as luciferase activity divided by β-gal activity to normalize transfection efficiency per microgram protein. The protein content in each sample was determined by the bicinchoninic acid (BCA) protein assay (Pierce Biotechnology, Rockford, IL, USA). In c-Src inhibitor experiment, the cells were treated PP2 or DMSO for 1 h after reporter genes transfection.

### 4.4. Immunoblot Analysis and Enzyme-Linked Immunosorbent Assay (ELISA)

The protein content in each sample was determined by bicinchoninic acid (BCA) protein assay. Proteins were fractionated on SDS-PAGE, transferred onto Hybond enhanced chemiluminescence nitrocellulose membranes (Amersham, Little Chalfont, UK), and probed with antibodies against Cx43 (Sigma-Aldrich), HIF-1α (Novus Biologicals, Littleton, CO, USA), c-Src (Cell Signaling Technology, Boston, MA, USA), phosphor-c-Src (Cell Signaling Technology) or monoclonal antibodies against β-actin (AC-15; Sigma-Aldrich). Horseradish peroxidase-conjugated goat anti-mouse IgG or anti-rabbit IgG (Jackson, West Grove, PA, USA) was used as the secondary antibody and protein-antibody complexes were visualized by enhanced chemiluminescence system (Amersham) [26,27]. The signals were quantified with ImageJ software (National Institutes of Health, Bethesda, MD, USA). The levels of mouse VEGF in supernatant of cells were determined by ELISA [17].

### 4.5. Assay of Endothelial Proliferation

HMEC-1 cells (2 × 10^3^/well) were cultured in 96-well plates overnight. The culture medium was then removed and replaced with conditioned medium. After 48 h, cell proliferation was assessed by the colorimetric WST-1 assay (Dojindo Labs, Tokyo, Japan) according to the manufacturer’s instructions [28,29].

### 4.6. Immunohistochemistry

To analyze microvessel density in the tumor sites, the whole tumors were excised and snap frozen at day 16. Frozen tumor sections were prepared with the aforementioned procedure, and incubated with rabbit anti-factor VIII-related antigen (DAKO, Carpinteria, CA, USA). After sequential incubation with appropriate peroxidase-labeled secondary antibody and aminoethyl carbazole (AEC) as substrate chromogen, tumor sections were counterstained with hematoxylin. Vessel density was determined by averaging the number of vessels in three areas of highest vessel density at ×400 magnification in each section [12,13].

### 4.7. Animal Studies

Groups of mice were subcutaneously (s.c.) inoculated with 10^6^ tumor cells. Palpable tumors were measured every 3 days in two perpendicular axes using a tissue caliper, and the tumor volumes were calculated as follows: (length of tumor) × (width of tumor)^2^ × 0.45.

### 4.8. Statistical Analysis

The unpaired, two-tailed Student’s *t* test was used to determine differences between groups. Any *p* value less than 0.05 is regarded statistically significant.

## 5. Conclusions

HIF-1α is frequently overexpressed in common tumors and there is a statistically significant correlation between the down-regulation of Cx43 and HIF-1α overexpression. Thus, increased HIF-1α activity resulting from loss of Cx43 expression may also contribute to the overexpression of VEGF, which is observed in our system tumor models.
